# Genomic profiling of methicillin-sensitive *Staphylococcus aureus* (MSSA) isolates in Kuwait hospitals

**DOI:** 10.3389/fmicb.2024.1361217

**Published:** 2024-07-17

**Authors:** Samar S. Boswihi, Wadha A. Alfouzan, Edet E. Udo

**Affiliations:** ^1^Department of Microbiology, College of Medicine, Kuwait University, Kuwait City, Kuwait; ^2^Microbiology Unit, Department of Laboratories, Farwaniya Hospital, Farwaniya, Kuwait

**Keywords:** MSSA, molecular genotyping, antibiotic resistance, *spa* types, DNA microarray

## Abstract

**Background:**

*Staphylococcus aureus* is an important pathogen that causes mild to invasive infections in hospitals and the community. Although methicillin-susceptible *Staphylococcus aureus* (MSSA) isolates continue to cause different infections, there is no data on the genetic backgrounds of the MSSA colonizing or causing infections in Kuwait hospitals. This study aimed to investigate MSSA isolated from patients admitted to Kuwait hospitals for antibiotic resistance and genetic backgrounds to understand their clonal composition.

**Methods:**

Consecutive MSSA isolates were collected from single patients during two surveillance periods in 2016 and 2021 in 13 public hospitals. The isolates were characterized using antibiogram, staphylococcal protein A (*spa*) typing, DNA microarray analysis, and multilocus sequence typing (MLST) using standard protocols.

**Results:**

A total of 446 MSSA was cultured from different clinical samples in 2016 (*n* = 240) and 2021 (*n* = 206). All isolates were susceptible to vancomycin [minimum inhibitory concentration (MIC) ≤ 2 mg/L], teicoplanin (MIC ≤2 mg/L), linezolid (MIC ≤4 mg/L), ceftaroline (MIC ≤2 mg/L), rifampicin, and mupirocin but were resistant to erythromycin (21.3%), clindamycin (14.0%), gentamicin (3.8%), kanamycin (10.5%), fusidic acid (27.0%), tetracycline (6.9%), trimethoprim (23.1%), and ciprofloxacin (35.2%). Molecular typing identified 155 *spa* types, dominated by t127 (15.0%), t084 (5.4%), t3841 (5.4%), t267 (2.4%), t442 (2.2%), t091 (2.2%), t021 (2.2%), and t003 (2.2%); 31 clonal complexes (CCs); and 56 sequence types (STs). The majority of the isolates (*n* = 265; 59.4%) belonged to CC1 (20.6%), CC15 (10.9%), CC22 (5.1%), CC30 (7.6%), CC361 (10.1%), and CC398 (4.7%).

**Discussion:**

The MSSA isolates belonged to diverse genetic backgrounds dominated by CC1, CC15, CC22, CC30, CC361, and CC398. The distribution of MSSA clones in 2016 and 2021 showed the stability of these clones over time. The study provides the first comprehensive data on the clonal distribution of MSSA in Kuwait hospitals.

## Introduction

*Staphylococcus aureus* is an opportunistic pathogen that causes healthcare-associated and community-associated infections. It is a common cause of nosocomial pneumonia, surgical site, bloodstream, cardiovascular, ear, eye, nose, and throat infections (Murray et al., 2012). Methicillin-susceptible *Staphylococcus aureus* (MSSA) is an important cause of infection that may result in mortality. Invasive infections caused by MSSA have been increasing in the United States (Arif et al., 2016; Crandall et al., 2020; Jackson et al., 2020) and Switzerland (Renggli et al., 2023). MSSA caused 78.6% of bloodstream infections in eight counties in the United States (Jackson et al., 2020) and 64.3% of bloodstream infections in children in Utah, United States (Crandall et al., 2020). Furthermore, Tinelli et al. (2009) reported outbreaks of skin and soft tissue infections caused by MSSA strains belonging to a PVL-producing sequence type (ST) 22. In addition, MSSA contributed to deaths resulting from cases of necrotizing fasciitis in the United States (Arif et al., 2016). Furthermore, MSSA has been associated with cases of postoperative and hematogenous meningitis. For example, Aguilar et al. (2010) reported that out of 12 cases of postoperative meningitis, 50% were caused by MSSA, and the other 50% were caused by MRSA, whereas out of 21 cases of hematogenous meningitis, 62% were caused by MSSA and 48% were caused by MRSA.

The ability of *S. aureus* strains to cause serious infections depends on their ability to express various virulence factors that allow them to adhere to and colonize cell surfaces, avoid the host’s immune system, and produce toxic effects on host tissues (Ferry et al., 2005; Gordon and Lowy, 2008; Murray et al., 2012). The virulence determinants of *S. aureus* include staphylococcal protein A (SpA), fibronectin-binding proteins A (FnbpA) and B (FnbpB), collagen-binding adhesin (Cna), and clumping factor A (ClfA) A and B (clfB), proteins that mediate bacterial attachment to the host cell. In addition, some strains of *S. aureus* secrete leucocidins, such as Panton-Valentine leukocidin (PVL), a pore-forming cytotoxin that causes leucocyte destruction and tissue necrosis and is associated with tissue necrosis of the skin and severe necrotizing pneumonia (Ferry et al., 2005). Some strains of *S. aureus* also produce pyrogenic toxins, such as staphylococcal enterotoxins, that cause fever and food poisoning (Jackson et al., 2020).

In addition to their ability to produce an outstanding armory of virulence factors, *S. aureus* also has the ability to easily acquire and transmit antibiotic resistance genes, which are located either on the bacterial chromosome or on mobile genetic elements (Lowy, 2003;Chambers and Deleo, 2009; Mlynarczyk-Bonikowska et al., 2022). Antibiotic resistance in *S. aureus* started with the acquisition of the *blaZ* gene, which encodes penicillinase, an enzyme that hydrolyzes benzylpenicillin (Chambers and Deleo, 2009; Mlynarczyk-Bonikowska et al., 2022). This was followed by the development of resistance to subsequently developed and clinically used antibiotics, including methicillin, a penicillinase-stable beta-lactam. Methicillin was introduced for clinical use in the late 1950s to treat infections caused by penicillinase-producing *S. aureus* (Chambers and Deleo, 2009). However, by 1961, *S. aureus* strains that were resistant to methicillin, known as methicillin-resistant *S. aureus* (MRSA), emerged (Jevons, 1961) due to the acquisition of the *mecA* gene that mediates the production of a novel penicillin-binding protein with a low affinity for binding to beta-lactams (Mlynarczyk-Bonikowska et al., 2022). MRSA strains are also resistant to all beta-lactam antibiotics except the fifth-generation cephalosporins. In addition, some MRSA strains are also resistant to multiple non-beta-lactam antibiotics, such as tetracycline mediated by *tet*(K), *te*t(M), erythromycin mediated by *erm*(A), *erm*(B), *erm*(C), *mph*(C), *msr*(A), chloramphenicol encoded by *cat* or *fexA*, aminoglycosides mediated by *aacA-aphD*, *aphA3*, or *aadD*, and fluoroquinolones mediated by *norA* or *gyrA* (Chambers and Deleo, 2009; Mlynarczyk-Bonikowska et al., 2022).

The MRSA isolates have been studied extensively in Kuwait for resistance to antibacterial agents, genetic backgrounds, and virulence profiles (Boswihi et al., 2016; Udo et al., 2016; Udo and Al-Sweih, 2017; Boswihi et al., 2020; Sarkhoo et al., 2021; Boswihi et al., 2022). In contrast, there is limited information on the antibiotic resistance, virulence profiles, and genotypes of MSSA-causing infections in Kuwait hospitals (Vali et al., 2017). Knowledge of the resistance patterns and genetic backgrounds of current *S. aureus* strains associated with infections and colonization can be helpful to clinicians, microbiologists, nurses, and infection control and prevention professionals, who make decisions on the treatment and prevention of *S. aureus* infections. Given the lack of information on the genotypes of *S. aureus* isolates circulating in Kuwait hospitals, this study aimed to determine the genotypes, antibiotic resistance, and virulence profiles of *S. aureus* obtained from patients in Kuwait hospitals. In addition, the study aimed to establish a genotype database for *S. aureus* in Kuwait, which will serve as a platform for further studies.

## Materials and methods

### *Staphylococcus aureus* strains

A total of 2,526 *S. aureus* were collected as a part of antibiotic susceptibility surveillance in the microbiology diagnostic service in 13 public hospitals during two 6-month periods in 2016 (1 February to 31 July; *n* = 1,668) and 2021 (1 April to 30 September; *n* = 858). The isolates were cultured from different clinical samples and identified in the diagnostic microbiology laboratories as *S. aureus* using standard bacteriological protocols, including Gram’s stain, growth on mannitol salt agar (Oxoid, Hampshire, United Kingdom), and positive tube coagulase and DNase tests. The isolates were identified as methicillin-resistant (MRSA) or methicillin-susceptible (MSSA) based on the results of antibiotic sensitivity testing using cefoxitin ETEST strips (bioMérieux, Marcy l’Étoile, France) and the *mecA* PCR (Zhang et al., 2005). A total of 446 MSSA isolates, confirmed by the absence of *mecA*, were investigated further in this study. Pure cultures of isolates on blood agar (Oxoid, Hampshire, United Kingdom) plates were submitted to the Gram-Positive Bacteria Research Laboratory, located in the Department of Microbiology, College of Medicine, Kuwait University, where the isolates were retested for purity and preserved in 40% glycerol (v/v in brain heart infusion broth) at −80°C for further analysis. Each isolate was from a single patient. The isolates were recovered by subculturing twice on brain heart infusion agar (Oxoid, Hampshire, United Kingdom) at 35°C for 24 h. Working stocks were preserved in 15% glycerol (v/v) at −20°C.

### Antimicrobial susceptibility testing (AST)

Antibiotic susceptibility testing was performed using the disk diffusion method against the following antibiotic disks (Oxoid, Hampshire, United Kingdom): benzylpenicillin (10 U), cefoxitin (30 μg), kanamycin (30 μg), mupirocin (200 μg), gentamicin (10 μg), erythromycin (15 μg), clindamycin (2 μg), chloramphenicol (30 μg), tetracycline (10 μg), trimethoprim (2.5 μg), fusidic acid (10 μg), rifampicin (5 μg), and ciprofloxacin (5 μg). Minimum inhibitory concentrations (MICs) for cefoxitin, vancomycin, teicoplanin, ceftaroline, daptomycin, and linezolid were determined using ETEST strips (bioMérieux, Marcy l’Étoile, France) according to the manufacturer’s instructions. *S. aureus* strains ATCC 25923 and ATCC 29213 were used for quality control of the disk diffusion and MIC determinations, respectively. The results were interpreted according to guidelines from the Clinical Laboratory Standard Institute (CLSI, 2020). Fusidic acid results were interpreted according to the European Committee on Antimicrobial Susceptibility Testing (EUCAST, 2023). The MSSA isolates that were resistant to three or more antimicrobial classes were classified as multidrug-resistant (MDR) (Magiorakos et al., 2012).

### DNA isolation

DNA isolation from *S. aureus* isolates was performed as described previously (Boswihi et al., 2016). Three to five colonies of overnight pure culture on brain heart infusion agar (Oxoid, Hampshire, United Kingdom) were picked and suspended in a lysing solution of 50 μL of lysostaphin (Sigma-Aldrich, Darmstadt, Germany) (150 μg/mL) and 10 μL of RNase (Sigma-Aldrich, Darmstadt, Germany) (10 μg/mL) in a sterile microfuge tube. The tube was incubated at 37°C in a heating block (Thermomixer, Eppendorf, Hamburg, Germany). After 20 min incubation, 50 μL of proteinase K (Sigma-Aldrich, Darmstadt, Germany) (20 mg/mL) and 150 μL of Tris buffer (0.1 M) were added to each tube and incubated further at 60°C in the heating block for 10 min. The tubes were then incubated at 95°C for 10 min and then centrifuged at 13,000 rpm for 5 min. The supernatant was transferred to a sterile microfuge tube and stored at 4°C until used for PCR.

### Molecular typing of *Staphylococcus aureus* strains

#### Staphylococcal protein A (*Spa*) typing

*Spa* typing was performed for all MSSA isolates. *Spa* typing was performed using primers and protocols published by Harmsen et al. (2003). *Spa* repeats were detected using the Ridom Staph Type software (Ridom GmbH, Wurzburg, Germany; https://www.ridom.de/staphtype), which subsequently assigned each isolate to a *spa* type.

### DNA microarray analysis

DNA microarray analysis was performed as described previously by Monecke et al. (2008) to assign each isolate to a clonal complex (CC) and determine antibiotic resistance genotypes and virulence determinants. These determinants include species markers, adhesions, genes encoding antibiotic resistance, leukocidins (*lukF-PV, lukS-PV, lukF, lukS, lukM, lukD, lukE, lukX, lukY*), hemolysins (*hl, hla, hlb, hlII*), exfoliative toxins (*etA, etB, etD*), enterotoxins (*sea, seb, sec, sed, see, seg, seh, sei, sej, sek, sel, selm, seln, selo, egc, seq, ser, selu*), biofilm encoding genes (*icaA, icaA, icaC, icaD, bap*), immune evasion complex (IEC) (staphylokinase, *sak*; chemotaxis-inhibiting protein, *chp*, and staphylococcal complement inhibitor, *scn*), arginine catabolic mobile element (ACME), accessory gene regulator alleles (*agrI, agrII, agrIII, agrIV*) and types of capsular polysaccharide (*cap1, cap5, cap8*). It also detects genes that identify SCC*mec* types. The technique was performed using the INTER-ARRAY Genotyping Kit *S. aureus* (Inter-Array GmbH, Bad Langensalza, Germany).

### Multilocus sequence typing (MLST)

Multilocus sequence typing (MLST) was performed on isolates representing different clonal complexes (CCs) and *spa* types. The technique involved the amplification and sequencing of seven housekeeping genes in each isolate (Enright et al., 2000; Tan et al., 2006). The housekeeping genes were sequenced in an automated 3130×1 genetic analyzer (Applied Biosystems, United States) following the manufacturer’s protocol. The sequences for each housekeeping gene were submitted to the MLST database at^1^ where the allelic profiles were generated, and the sequence type (ST) was assigned.

## Results

A total of 446 MSSA were isolated in two 6-month periods in 2016 (1 February to 31 July; *n* = 240) and 2021 (1 April to 30 September, *n* = 206). Most of the isolates were obtained from skin and soft tissue infections (*n* = 129), nasal swabs (*n* = 66), and high vaginal swabs (HVSs) (*n* = 58). The remaining isolates were obtained from blood (*n* = 37), sputum (*n* = 22), endotracheal aspirate (*n* = 35), urine (*n* = 18), throat (*n* = 16), eye (*n* = 13), ear (*n* = 10), fluid (*n* = 8), tissue (*n* = 6), groin (*n* = 6), and umbilical stump (*n* = 2). One isolate each was obtained from a catheter exit site, bronchoalveolar lavage, and axillar swab. The sources of 17 isolates were not provided.

### Antibiotic susceptibility of the MSSA isolates

All 446 isolates were sensitive to cefoxitin (MIC ≤2 mg/L), which indicated that the isolates were methicillin-sensitive *Staphylococcus aureus* (MSSA), vancomycin (MIC ≤2 mg/L), teicoplanin (MIC ≤2 mg/L), linezolid (MIC ≤4 mg/L), ceftaroline (MIC ≤2 mg/L), but were resistant to penicillin G (*n* = 249; 55.8%), gentamicin (*n* = 17; 3.8%), kanamycin (*n* = 47; 10.5%), erythromycin (*n* = 95; 21.3%), clindamycin-inducible resistance (*n* = 57; 12.7%), clindamycin-constitutive resistance (*n* = 6; 1.3%), chloramphenicol (*n* = 3; 0.7%), tetracycline (*n* = 31; 6.9%), trimethoprim (*n* = 103; 23.1%), fusidic acid (*n* = 121; 27.0%), ciprofloxacin (*n* = 157; 35.2%), rifampicin (*n* = 1; 0.2%), and low-level resistance mupirocin (*n* = 2; 0.4%). Two isolates showed reduced susceptibility to daptomycin (MIC 1.5 mg/L). In total, 106 (23.8%) of the isolates showed multidrug resistance to antibiotics.

### Antibiotic resistance genotypes of the MSSA isolates

All penicillin-resistant isolates tested positive for the penicillin resistance operon *blaZ/blal/blaR*, while the gentamicin-resistant isolates tested positive for *aacA-aphD*. Isolates resistant to kanamycin but susceptible to gentamicin harbored *aphA3* or *aadD,* while the fusidic acid-resistant isolates were positive for *fusC* or *fusB*. The erythromycin and clindamycin-resistant isolates tested positive for *erm*(A) (*n* = 5) and *erm*(C) (*n* = 35), those resistant to erythromycin but susceptible to clindamycin carried *msr*(A) (*n* = 24) and *mph*(C) (*n* = 20), and those resistant to clindamycin but susceptible to erythromycin harbored *lnu*(A) (*n* = 9). The trimethoprim-resistant isolates harbored *dfrS1*, while the tetracycline-resistant isolates carried *tet*(K). The distribution of antibiotic resistance genotypes of the isolates is presented in Table 1.

**Table 1:** Distribution and characteristics of the dominant MSSA clones in 2016 and 2021.

Strain-microarray (N)	2016	2021	Antibiotic resistance genes (N)	Toxin genes (N)	Miscellaneous genes (N)
**Clonal complex 1 (84)**					
CC1-MSSA (5)	3	2	*blaZ, blaI, blaR*	*sea* (1), *seb* (1), *sec* (1), *seh* (5), *sek* (1), *seq* (1), *sel* (1)	*agrIII, cap8, sak* (3), *scn* (3), *icaA/C/D*
CC1-MSSA [PVL+] (1)	1		*blaZ, blaI, blaR*	PVL, *sea, seb, seh, sek, seq*	*agrIII, cap8, sak, scn, icaA/C/D*
CC1-MSSA-[ccrAB1] (2)		2	*ermC, sdrM*	*sea* (1), *seh* (2), *sek* (1), *seq* (1)	*agrIII, cap8, sak* (1), *scn* (1), *icaA/C/D, ccrA-1, ccrB-1*
CC1-MSSA-[fus + ccrAB1] (2)		2	*blaZ, blaI, blaR, fusC*	*sea* (1), *seh* (2), *sek* (2), *seq* (1)	*agrIII, cap8, sak, scn, icaA/C/D, ccrA-1, ccrB-1*
CC1-MSSA-SCC*fus* (72)	34	38	*blaZ, blaI, blaR, erm*(C) (22), *msr*(A) (9), *mph*(C) (9), *aphA3* (14), *sat* (13), *fusC* (72)	*sea* (64), *seb* (25), *seh* (72), *sek* (67), *seq* (67), *ser* (1), *sej* (1)	*agrIII, cap8, sak* (70), *scn* (70), *icaA/C/D, ccrA-1 (70), ccrB-1* (72), *ccrB-3* (18), *Q9XB68-dcs* (1)
CC1-MSSA-SCC*fus* [PVL+] (2)	1	1	*blaZ, blaI, blaR, fusC, aphA3* (1)	PVL, *sea, seh, sek, seq*	*agrIII, cap8, sak, scn, icaA/C/D, ccrA-1, ccrB-1, ccrB-3* (1)
ST573/772-MSSA (3)	1	2	*blaZ, blaI, blaR*	*sec, sel, egc*	*agrII, cap5, scn, icaA/C/D*
ST573/772-MSSA [PVL+] (5)	4	1	*blaZ, blaI, blaR, msr*(A) (3), *mph(C)* (3), *aphA3* (3), *sat* (3)	PVL, *tst* (1), *sea, seb* (1), *sec, sel, egc*	*agrII, cap5, scn, icaA/C/D*
**Clonal complex 15 (49)**					
CC15-MSSA (48)	28	20	*blaZ, blaI, blaR, erm*(C) (2), *lnu*(A) (8), *aadD* (11), *tet*(K) (9), *msr*(A) (1), *mph*(C) (1), *fusC* (2), *fusB* (1), *cat* (1)	*etA* (2), *tst* (2), *seb* (8), *sed* (1), *sej* (1), *sek* (1), *seq* (1), *ser* (1)	*agrII, cap8, sak, scn, icaA/C/D*
CC15-MSSA [PVL+] (1)	1		*blaZ, blaI, blaR,*	PVL, *sek, seq*	*agrII, cap8, sak, scn, icaA/C/D*
**Clonal complex 22 (23)**					
CC22-MSSA (18)	13	5	*blaZ, blaI, blaR*	*tst* (11), *sec* (2), *sel* (3), *egc*	*agrI, cap5, sak* (16), *chp* (14), *scn* (16), *icaA/C/D*
CC22-MSSA [ccrA/B-4+] (1)	1		*blaZ, blaI, blaR, fusC*	*tst, egc*	*agrI, cap5, sak, chp, scn, icaA/C/D, ccrA-4*
CC22-MSSA [PVL+] (4)	4		*blaZ, blaI, blaR, aacA-aphD* (2), *dfrS1* (2)	PVL, *tst* (1)	*agrI, cap5, sak, chp, scn, icaA/C/D*
**Clonal complex 30 (34)**					
CC30-MSSA (17)	10	7	*blaZ, blaI, blaR, erm*(A) (5), *msr*(A) (1), *tet*(K) (1)	*tst* (12), *sea* (9), *egc*	*agrIII, cap8, sak* (16), *chp* (14), *scn* (16), *icaA/C/D*
CC30-MSSA [PVL+] (13)	10	3	*blaZ, blaI, blaR, msr*(A) (1), *lnu(A)* (1), *aadD* (3), *tet*(K) (3), *mupA* (1)	*PVL, tst* (3), *sea* (7), *egc*	*agrIII, cap8, sak, chp, scn, icaA/C/D*
CC30-MSSA [truncated egc] (1)		1	*blaZ, blaI, blaR, msr*(A)	*tst, egc*	*agrIII, cap8, icaA/C/D*
ST34/42-MSSA (3)	1	2	*blaZ, blaI, blaR*	*tst, seh, egc*	*agrIII, cap8, sak, scn, icaA/C/D*
**Clonal complex 88 (19)**					
CC88-MSSA (5)	3	2	*blaZ, blaI, blaR, cat* (1)	*sea* (3), *seb* (1), *sek* (1), *seq* (1)	*agrIII, cap8, sak, chp, scn, icaA/C/D*
CC88-MSSA [PVL+] (14)	6	8	*blaZ, blaI, blaR, erm*(C) (4), *aadD* (1), *tet*(K) (3)	PVL*, sea* (13), *seb* (1), *sek* (1), *seq* (1)	*agrIII, cap8, sak, chp, scn, icaA/C/D*
**Clonal complex 121 (13)**					
CC121-MSSA (9)	2	7	*blaZ, blaI, blaR, erm*(C) (2)	*etA, etB* (5), *seb* (1), *egc*	*agrIV, cap8, sak, scn, icaA/C/D, edinC* (6)
CC121-MSSA [PVL+] (4)	3	1	*blaZ, blaI, blaR, erm*(C) (1)	PVL, *seb* (3), *egc*	*agrIV, cap8, sak, scn, icaA/C/D*
**Clonal complex 361 (45)**					
CC361-MSSA (44)	24	20	*blaZ, blaI, blaR, erm*(C) (3), *msr*(A) (7), *mph*(C) (6), *aadD* (2), *aphA3* (10), *sat* (10), *fusB* (1), *tet*(K) (1), *qacA* (2)	PVL (3), *tst* (1), *sea* (4), *seb* (1), *sek* (1), *seq* (1), *egc*	*agrI, cap8, sak* (41), *chp (12), scn* (41), *icaA/C/D*
CC361-MSSA [PVL+] (1)	1			PVL, *egc*	*agrI, cap8, sak, chp, scn, icaA/C/D*
**Clonal complex 398 (22)**					
CC398-MSSA (8)	1	7	*blaZ, blaI, blaR*	*sea* (1)	*agrI, cap5, chp, scn, icaA/C/D*
ST291/813-MSSA (11)	4	7	*blaZ, blaI, blaR, msr*(A) (1), *mph*(C) (1), *aadD* (1)	*etD*	*agrI, cap5, sak, chp, scn, icaA/C/D, edinB*
ST291/813-MSSA [PVL+] (3)	3		*blaZ, blaI, blaR, tet*(K) (1)	PVL, *etD*	*agrI, cap5, sak, chp, scn, icaA/C/D, edinB*

*aacA-aphD*, aminoglycoside adenyl−/phosphotransferase; *aadD,* aminoglycoside adenyl transferase; *aphA3*, aminoglycoside phosphotransferase; *cat*, chloramphenicol acetyl transferase; *fexA*, chloramphenicol/florfenicol exporter; *dfrS1*, dihydrofolate reductase mediating trimethoprim resistance; *erm(C)*, rRNA methyltransferase (C); *msr(A)*, macrolide efflux pump; *mph(C)*, macrolide phosphotransferase; *fusC*, fusidic acid resistance gene (Q6GD50); *fusB*, fusidic acid resistance gene (=far1); *mupA*, isoleucyl-tRNA synthetase associated with mupirocin resistance; *tet(K)*, tetracycline efflux protein; *sat*, streptothricin acetyltransferase; *lnu(A)*, lincosamide nucleotidyltransferase (=linA); *qacA*, multidrug efflux protein A; *sak*, staphylokinase; *chp*, chemotaxis-inhibiting protein; *scn*, staphylococcal component inhibitor; *egc*; enterotoxin gene cluster (seg/i/selm/n/o/u); PVL, Panton Valentine leucocidin; *etA*; exfoliative toxin serotype A; *etB*, exfoliative toxin serotype B; *etD*, exfoliative toxin D; *icaA/C/D*, intercellular adhesion protein A,C,D; *edinB*, epidermal cell differentiation inhibitor B; *edinC*, epidermal cell differentiation inhibitor C; *ccrA-1*, cassette chromosome recombinase genes A-1; *ccrA-4*, cassette chromosome recombinase gene A-4; *ccrB-1*, cassette chromosome recombinase genes B-1; *ccrB-3*, cassette chromosome recombinase gene B-3; Q9XB68-dcs, hypothetical protein from SCCmec elements; *agr*, accessory gene regulator; *cap*, capsular polysaccharide.

### Molecular characteristics of the MSSA isolates

Molecular typing characterized the MSSA isolates into 155 *spa* types, 31 CCs, and 56 STs. The most prevalent *spa* types, which constituted 31.8% of all isolates, were t127 (*n* = 67), t084 (*n* = 24), t3841 (*n* = 24), t267 (*n* = 11), t442 (*n* = 10), t091 (*n* = 10), t021 (*n* = 10), and t003 (*n* = 10). The remaining *spa* types, detected in less than 10 isolates, are summarized in Table 2. The *spa* types could not be assigned to 19 isolates because no amplified products were obtained using the current set of primers on repeated testing.

**Table 2:** Distribution of spa types among MSSA isolates.

*Spa* types	The number of isolates carrying each *spa* types
t127	67
t084, t3841	24
t267	11
t003, t021, t091, t442	10
t159, t189, t2393	8
t903, t1149	7
t223	6
t008, t346, t937, t1451	5
t012, t131, t304, t380, t491, t693, t948, t2016	4
t005, t309, t315, t359, t360, t888,	3
t015, t019, t105, t148, t164, t184, t2119, t213, t254, t311, t314, t345, t349, t355, t559, t657, t701, t1309, t1508, t10135, t1985, t2085, t5163, t6205	2
t014, t018, t024, t044, t073, t144, t167, t177, t186, t230, t249, t279, t310, t318, t330, t342, t363, t384, t448, t486, t571, t591, t608, t617, t645, t668, t706, t729, t774, t817, t861, t864, t945, t991, t1057, t1193, t1227, t12370, t12758, t12893. t1427, t14472, t1458, t1517, t16061, t1614, t1676, t16945, t1727, t1784, t1839, t1877, t18998, t20727*, t20728*, t20729*, t20730*, t20731*, t20732*, t20733*, t20734*, t20735*, t20736*, t2078, t20812*,t2235, t2251, t2473, t2509, t2526, t2726, t2767, t2868, t2883, t3092, t3095, t3242, t3243, t3338, t3364, t3564, t3689, t3864, t4333, t4407, t4522, t4672, t4714, t5229, t5598, t5919, t6101, t6702, t7164, t7656, t7766, t8263, t9231, t9411	1

*Novel *spa* types.

Among the 31 CCs, the dominant lineages were CC1/ST573/ST772 (*n* = 92), CC15 (*n* = 49), CC361 (*n* = 45), CC30/ST34/ST42 (*n* = 34), CC22 (*n* = 23), and CC398/ST291/ST813 (*n* = 22), which constituted 59.4% of the MSSA isolates. The other CCs were CC88 (*n* = 19), CC5 (*n* = 18), CC97 (*n* = 15), CC7 (*n* = 13), CC121 (*n* = 13), CC8/ST239/ST72 (*n* = 11), CC188 (*n* = 11), CC45 (*n* = 9), CC96 (*n* = 8), CC6 (*n* = 8), CC1153 (*n* = 7), CC2250/2277 (*n* = 6), CC25 (*n* = 5), CC12 (*n* = 4), CC1290 (*n* = 4), CC101 (*n* = 3), and CC1223 (*n* = 3). CCs identified in two isolates each included CC20, CC152, CC9/ST834, and CC2990, whereas CC80, CC707, CC913, and CC1156 were each identified in a single isolate. ST2867 was detected in eight isolates, whereas ST1303, ST2081, ST2479, and ST7985 were each detected in a single isolate.

The 105 isolates selected for MLST based on CC and *spa* types identified 56 STs. The distribution of STs identified by MLST is discussed below, along with the molecular characteristics of each CC. The distribution of the dominant CCs and virulence genes of the isolates is summarized in Table 1. The detailed distribution of species markers and genes encoding antibiotic resistance and virulence factors are presented in Supplementary Table S1. The molecular characteristics of the isolates are presented below.

#### Clonal complex 1

In total, 92 MSSA isolates were identified as CC1 (*n* = 84) and ST573/ST772 (single variant of ST1) (*n* = 8) and consisted the following genotypes: CC1-MSSA-SCC*fus* (*n* = 72), CC1-MSSA (*n* = 5), CC1-MSSA-[ccrAB1] (*n* = 2), CC1-MSSA-[fus + ccrAB1] (*n* = 2), CC1-MSSA-SCC*fus* [PVL^+^] (*n* = 2), CC1-MSSA [PVL^+^] (*n* = 1), ST573/ST772-MSSA (*n* = 3), and ST573/ST772-MSSA [PVL^+^] (*n* = 5). A total of 13 *spa* types were identified among CC1-MSSA isolates. These included t127 (*n* = 67), t948 (*n* = 4), t559 (*n* = 2), t1508 (*n* = 2), t177 (*n* = 1), t591 (*n* = 1), t1784 (*n* = 1), t2016 (*n* = 1), t3564 (*n* = 1), t3864 (*n* = 1), t12758 (*n* = 1), t14472 (*n* = 1), and t18998 (*n* = 1). All nine CC1 representative isolates selected for MLST analysis belonged to ST1.

A total of 78 CC1 isolates harbored combinations of the cassette chromosome recombinase (*ccr*) genes *ccrA-1, ccrB-1,* and *ccrB-3,* mostly in isolates carrying SCC*fusC*, a fusidic acid resistance determinant, which has been detected in the MRSA isolates (Monecke et al., 2023). The eight ST573/ST772-MSSA isolates were associated with *spa* types t345 (*n* = 2), t657 (*n* = 2), t1839 (*n* = 1), and t2085 (*n* = 1), and a novel *spa* type t20736 (*n* = 1). One ST573/ST772 isolate could not be assigned to a *spa* type. Four representatives of ST573/ST772-MSSA isolates selected for MLST belonged to ST772 (*n* = 2), ST573 (*n* = 2), and ST3206 (*n* = 1) (1–1–1-1-4-4-1; the double locus variant of ST1).

#### Clonal complex 5

A total of 18 MSSA isolates belonged to CC5 and were identified as CC5-MSSA (*n* = 16), CC5-MSSA[PVL^+^] (*n* = 1), and CC5-MSSA-[fus + ccrC] (*n* = 1). The 18 isolates belonged to six *spa* types, with t442 (*n* = 10) as the most common. The other *spa* types were t311 (*n* = 2), t105 (*n* = 2), and t9231, t8263, and t668 detected in single isolates. All three representative CC5-MSSA isolates selected for MLST belonged to the same ST, ST5. Two of the fusidic acid-resistant isolates carrying *fusC* also carried the *ccr* genes, *ccrC* (Supplementary Table S1).

#### Clonal complex 7

The CC7-MSSA consisted of 13 isolates classified into five *spa* types: t091 (*n* = 8), t3338 (*n* = 1), t360 (*n* = 1), t7164 (*n* = 1), and a novel *spa* type t20734 (*n* = 1). Three representative isolates selected for MLST belonged to the same ST, ST789. The ST789 (allele profile = 3–4–1-4-4-6-3) is a single locus variant of ST7 (allele profile = 5–4–1-4-4-6-3).

#### Clonal complex 8

A total of 11 CC8-MSSA isolates were identified as CC8-MSSA (*n* = 5), CC8-MSSA [PVL^+^] (*n* = 1), CC8-MSSA-SCC*fus* (*n* = 1), ST72-MSSA (*n* = 3), and ST239-MSSA (*n* = 1). The majority of the PVL-negative and PVL-positive CC8-MSSA isolates (*n* = 5) belonged to the *spa* type t008. An MLST analysis of four representative CC8-MSSA isolates assigned them to ST8 (*n* = 3; allele profile = 3–3–1-1-4-4-3) and one novel ST, ST8659 (allele profile = 1,003-3-1-1-4-4-3), which is a single locus variant of ST8. The ST72-MSSA isolates belonged to ST72.

All CC8 isolates were positive for *agrI* and cap5 but varied in the carriage of *sak, scn*, and *chp*. The three ST72-MSSA isolates harbored *egc*, which was absent in the ST8 and ST239 isolates.

#### Clonal complex 15

In total, 49 isolates belonged to CC15-MSSA. One isolate was positive for PVL and identified as CC15-MSSA [PVL^+^]. The isolates belonged to 15 *spa* types dominated by t084 (n = 24) followed by t346 (*n* = 5), t491 (*n* = 4), t184 (*n* = 2), t254 (*n* = 2), t2119 (*n* = 2), t10135 (*n* = 2), t144 (*n* = 1), t279 (*n* = 1), t360 (*n* = 1), t774 (*n* = 1), t1517 (*n* = 1), t1727 (*n* = 1), t4714 (*n* = 1), and t12370 (*n* = 1). Six CC15-MSSA isolates were selected for MLST. Five of the PVL-negative isolates belonged to ST15, and one novel ST, ST8656 (allele profile = 13–13–1-1-12-948–13), while the PVL-positive isolate belonged to ST199 (allele profile = 13–13–1-1-12-1-13). The ST199 and ST8656 are single locus variants of ST15 (allele profile = 13–13–1-1-12-11–13).

#### Clonal complex 22

A total of 23 isolates were identified as CC22-MSSA and were classified into three genotypes, namely, CC22-MSSA (*n* = 18), CC22-MSSA [PVL^+^] (*n* = 4), and CC22-MSSA [ccrA/B-4^+^] (*n* = 1) and 14 *spa* types. The *spa* types were t223 (*n* = 6), t005 (*n* = 3), and t309 (*n* = 3), and each of t608, t3242, t3243, t6101, t310, t2235, t3689, t9411, t2251, t249, and t16061. All of the five representative CC22 isolates selected for MLST were ST22. The fusidic acid resistance gene, *fusC,* was found in the CC22-MSSA [ccrA/B-4^+^] isolate, which also harbored the *ccrA4* gene.

#### Clonal complex 30

In total, 34 CC30-MSSA isolates were classified into four genotypes: CC30-MSSA (*n* = 17), CC30-MSSA [PVL^+^] (*n* = 13), CC30-MSSA [truncated *egc*] (*n* = 1), and ST34/ST42-MSSA (*n* = 3). The isolates belonged to 17 *spa* types: t021 (*n* = 10), t012 (*n* = 4), t019 (*n* = 2), t018 (*n* = 1), t318 (*n* = 1), t342 (*n* = 1), t363 (*n* = 1), t486 (*n* = 1), t617 (*n* = 1), t1676 (*n* = 1), t2509 (*n* = 1), t2726 (*n* = 1), t2868 (*n* = 1), t3095 (*n* = 1), t4672 (*n* = 1), and—two novel *spa* types—t20727 and t20729. One isolate could not be assigned to a *spa* type. The six CC30-MSSA representative isolates selected for MLST belonged to ST30 (allele profile = 2–2–2-2-6-3-2) and one novel ST, ST8664 (allele profile = 2–2–2-2-6-624-2), which is single locus variant of ST30. The three ST34/ST42-MSSA isolates were classified into three different *spa* types (t864, t817, and t1057) and two STs (ST34 and ST2693).

#### Clonal complex 88

A total of 19 CC88-MSSA isolates were classified into CC88-MSSA (*n* = 5) and CC88-MSSA [PVL^+^] (*n* = 14). The CC88-MSSA belonged to 10 *spa* types and 2 STs. The dominant *spa* type was t2393 (*n* = 8), followed by t5163 (*n* = 2), t693 (*n* = 2), and each of t186, t448, t729, t2526. t4333, t5919, and t7656. The two STs, namely, ST88 (*n* = 2) (allele profile = 22–1–14-23–12-4-31) and ST2884 (*n* = 2) (allele profile = 22–1–14-220-12-4-31; single locus variant of ST88), were identified among the four representative CC88 isolates that were selected for MLST.

#### Clonal complex 97

The 15 CC97-MSSA isolates belonged to four *spa* types and one ST (ST97). The *spa* type t267 (*n* = 10) was the dominant type, followed by t359 (*n* = 3) and t693 (*n* = 1). One isolate could not be assigned a *spa* type. All isolates were positive for *agrI* and *cap5* but varied in the carriage of *sak* and *scn* (Table 1).

#### Clonal complex 121

In total, 13 MSSA isolates belonged to CC121 and were identified as CC121-MSSA (*n* = 9) and CC121-MSSA [PVL^+^] (*n* = 4) and were assigned to four *spa* types: t159 (*n* = 8), t314 (*n* = 2), t645 (*n* = 1) and t1193 (*n* = 1). One isolate could not be assigned to a *spa* type. Three representative isolates selected for MLST were identified as ST120 (allele profile: 6–5–6-2-7-14-2), ST837 (allele profile: 6–5–6-2-7-14–3), and one novel ST, ST8667 (allele profile = 6–5–6-2-162-14–3), which are single locus variants of ST121 (allele profile = 6–5–6-2-7-14-5). The isolates differed in their exfoliative toxin gene profiles (Table 1).

#### Clonal complex 188

A total of 11 CC188-MSSA isolates were detected and assigned to four *spa* types, t189 (*n* = 8), t693 (*n* = 1), t2883 (*n* = 1), and t5229 (*n* = 1), and two STs, ST188 (allele profile = 3–1–1-8-1-1-1) and ST8741 (novel ST, allele profile = 3–1–1-8-1-1-1137). The isolates varied in the carriage of *chp* and enterotoxin genes (Table 1).

#### Clonal complex 361

In total, 45 isolates were identified as CC361-MSSA (*n* = 44) and CC361-MSSA [PVL^+^] (*n* = 1). The isolates were assigned to 10 *spa* types, with 75.5% of them belonging to t3841 (*n* = 24). The other *spa* types were t003 (*n* = 10), t315 (*n* = 3), t1309 (*n* = 2), and each of t1427, t16945, t1227, t014, t384, and a novel *spa* type, t20812. The six representative isolates selected for MLST were assigned to ST672 (*n* = 5) and ST361 (*n* = 1). The CC361-MSSA isolates varied in the carriage of genes encoding enterotoxins and immune evasion clusters (Table 1).

#### Clonal complex 398

A total of 22 isolates were identified as CC398-MSSA (*n* = 8), ST291/ST813-MSSA (*n* = 11), and ST291/ST813-MSSA [PVL^+^] (*n* = 3). *Spa* typing identified t1451 (*n* = 5), t1985 (*n* = 2) and t571 (*n* = 1) among the CC398-MSSA isolates, while t1149 (*n* = 7), t937 (*n* = 5), t1614 (*n* = 1), and t5598 (*n* = 1) were identified among the PVL-negative and PVL-positive ST291/ST813-MSSA isolates. Four representative isolates selected for MLST were classified as ST398 (*n* = 2), ST291 (*n* = 1), and ST3629 (*n* = 1). Two isolates were assigned to novel STs, ST8660 (allele profile: 159–35–19-2-20-26–39) and ST8666 (allele profile: 3–37–19-2-20-26-1139).

#### Other clonal complexes

In total, 18 CCs and five STs were detected in less than 10 MSSA isolates. These were CC45 (*n* = 9), CC6 (*n* = 8), CC96 (*n* = 8), ST2867 (*n* = 8), CC1153 (*n* = 7), CC2250/2277 (*n* = 6), CC25 (*n* = 5), CC12 (*n* = 4), CC1290 (*n* = 4), CC101 (*n* = 3), CC20 (*n* = 2), CC152 (*n* = 2), CC1223 (*n* = 3), CC2990 (*n* = 2), CC9/ST834 (*n* = 2), CC80 (*n* = 1), CC707 (*n* = 1), CC913 (*n* = 1), CC1156 (*n* = 1), ST1303 (*n* = 1), ST2081 (*n* = 1), ST2479 (*n* = 1), and ST7985 (*n* = 1).

The nine CC45 isolates were identified as CC45-MSSA (*n* = 8) and CC45-MSSA [egc deletion variants] (*n* = 1). The isolates were assigned to six *spa* types, including t015 (*n* = 2), t230 (*n* = 1), t073 (*n* = 1), t861 (*n* = 1), t330 (*n* = 1), and t706 (*n* = 1). Two of the isolates could not be assigned to *spa* types. Four representative isolates belonged to ST45 (*n* = 2), ST508 (*n* = 1), and a novel ST8740 (allele profile: 1004–40–8-6-10-3-2; the double locus variant of ST45).

Eight isolates were identified as CC6-MSSA and assigned to four *spa* types: t304 (*n* = 4), t701 (*n* = 2), t4407 (*n* = 1), and a novel type, t20731 (*n* = 1). All three representative isolates belonged to ST6.

The six CC1153-MSSA isolates belonged to the same *spa* type, t903, and two STs, ST1153 (*n* = 5) and—a novel ST—ST8657 (*n* = 1) (allele profile: 1002–13–1-1-124-5-3; a single locus variant of ST1153). One isolate was positive for PVL (Supplementary Table S1).

Six isolates were identified as CC2250/2277, which is a *Staphylococcus argenteus*-like strain. None of the isolates were assigned to a *spa* type on repeated testing. The isolates were also negative for *cap, agr,* and *coa,* which encode capsular polysaccharide, accessory gene regulator, and coagulase, respectively.

The eight ST2867-MSSA isolates belonged to six *spa* types: t2016 (*n* = 3) and each of t148, t20730, t20732, t20733, and t20735. Two of the isolates identified by microarray analysis as ST2867-MSSA isolates were assigned to a novel ST, ST8662 (allele profile: 1–152–1-8-1028-5-11), by MLST.

The eight isolates identified as CC96-MSSA defined a single ST, ST96, but belonged to four *spa* types, t380 (*n* = 4) and each of t360, t267, and t2085. One isolate was not assigned to a *spa* type.

Five isolates were identified as CC25-MSSA. The isolates belonged to a single ST, ST25, and three *spa* types, t349 (*n* = 2), t6205 (*n* = 2), and t167 (*n* = 1).

The molecular characteristics of CC12, CC1290, CC101, CC20, CC152, CC1223, CC2990, CC80, CC707, CC913, CC1156, CC9/ST834, ST1303, ST2081, ST2479, and ST7985 are summarized in Supplementary Table S1.

### A comparison of MSSA clones obtained in 2016 and 2021

The distribution of the MSSA isolates in 2016 and 2021 was compared to observe whether there were changes in the composition and prevalence of the MSSA clones. The results summarized in Table 1 showed that 27 CCs were present in both years. Six CCs and singletons that were isolated only in 2016 include CC707, CC2250/2277, ST834, ST1303, ST2479, and ST239, while the CCs and singletons detected only in 2021 include CC9, CC101, CC1153, CC12, CC2990, CC80, CC913, ST2081, and ST7985.

Overall, the dominant CCs were CC1, CC15, CC361, CC30, CC22, and CC88, which together constituted 56.2% of the MSSA isolates. The 33 CCs obtained in 2016 were slightly lower than the 36 CCs obtained in 2021. The number of the MSSA isolates belonging to CC1, CC88, CC121, CC45, CC188, CC25, CC1223, CC398, and ST2867 were higher in 2021 than in 2016. In contrast, the number of isolates belonging to CC5, CC7, CC97, CC8, CC1153, CC15, CC361, CC30, CC22, and CC1290 were lower in 2021 compared to 2016.

## Discussion

This study provides the first detailed report on the molecular characteristics of clinical MSSA isolates in Kuwait hospitals. The isolates were susceptible to vancomycin, teicoplanin, linezolid, and ceftaroline. However, two isolates demonstrated reduced susceptibility to daptomycin (MIC: 1.5 μg/mL). Although daptomycin non-susceptibility in *S. aureus* (MIC: >1 μg/mL) has been reported following treatment with the antibiotic in Taiwan and Italy (Kuo et al., 2009; Lee et al., 2010; Sabat et al., 2018), this is the first report of daptomycin non-susceptibility in MSSA in Kuwait. Unfortunately, there is no information on whether the patients whose isolates were daptomycin insensitive had been treated with daptomycin, which would have indicated whether the phenotype was due to direct antibiotic use. Nevertheless, clinical microbiologists should be alerted by this development to prevent the early emergence of resistance and transmission.

The MSSA isolates belonged to 31 CCs and 155 *spa* types, with 10 CCs comprising CC1/ST573/ST772 (*n* = 92), CC15 (*n* = 49), CCC361 (*n* = 45), CC30 (*n* = 34), C22 (*n* = 23), while CC398/ST291/ST813 (*n* = 22) constituting 59.4% (*n* = 265) of the isolates. The remaining 182 isolates (40.8%) belonged to 26 CCs that occurred sporadically. The dominant CCs, CC1, CC15, CC22, CC30, and CC361, were similar to the dominant CCs among clinical MRSA populations in Kuwait hospitals (Boswihi et al., 2016, 2020). However, differences were observed in the proportions of the dominant CCs among the MSSA in this study and those of MRSA isolates studied previously (Boswihi et al., 2016, 2020). In contrast, CC1 was the dominant CC, and CC5 was obtained sporadically in this study; CC5 constituted the dominant CC among MRSA isolates in Kuwait (Boswihi et al., 2020). In addition, ST239—the healthcare-associated MRSA clone—CC6, and CC80, which were dominant among MRSA populations in Kuwait hospitals (Boswihi et al., 2016), were detected only sporadically in this study, suggesting that these MSSA isolates were probably acquired independently of their corresponding local MSSA strains. The study also revealed the presence of clones, such as CC12, CC20, CC25, and CC1156, and many of the uncommon CCs among the MSSA isolates that have not been detected among the MRSA population in Kuwait hospitals to date, suggesting a greater diversity among the MSSA populations.

It is interesting that the major CC1-MSSA genotype—CC1-MSSA-SCC*fus*—belonged to *spa* type—t127—and included variants that harbored the SCC*fus* composite genetic element, the *ccr* genes, *ccrAB1*. This gene is associated with the SCC*mec* element and methicillin resistance (Monecke et al., 2023), similar to the characteristics of CC1-MRSA-IV-SCC*fus* isolates that were reported previously in Kuwait (Boswihi et al., 2020) and elsewhere (Monecke et al., 2023). CC1 has also been recognized previously in both MSSA and MRSA isolates (Deurenberg and Stobberingh, 2008; Breurec et al., 2011; Aggarwal et al., 2019). However, the presence of similar enterotoxin gene profiles in both CC1-MSSA and CC1-MRSA carrying the SCC*fus* element and the presence of *ccrAB1* in the CC1-MSSA-SCC*fus* and CC1-MSSA-[ccrAB1] isolates suggested that these MSSA isolates might be the precursors of the CC1-MRSA-SCC*fus* isolates. Alternatively, the CC1-MSSA-SCC*fus* isolates might have emerged from corresponding MRSA strains following the deletion of the *mecA* gene. The *mecA*-deleted strain (CC1-MSSA-SCC*fus*) was then spread successfully in different hospitals. A comparison of CC1-MRSA-SCC*fus* and CC1-MSSA-SCC*fus* isolates using whole-genome sequencing would help clarify this issue.

The CC15-MSSA in this study shares similarities and differences with CC15-MRSA isolates previously reported in Kuwait hospitals (Udo et al., 2020). The CC15-MSSA isolates were positive for *agrII, cap8, chp,* and *scn,* and most belonged to the *spa* type t084, similar to that reported for the CC15-MRSA+SCC*fus* isolates (Udo et al., 2020). However, some of the CC15-MSSA isolates (12/49; 24.5%), in this study, variably harbored *seb*, *sed, sej*, *sek*, *seq*, *ser, selm, eta*, *tst1,* and PVL, whereas the CC15-MRSA isolates obtained in Kuwait hospitals (Udo et al., 2020) and elsewhere (Monecke et al., 2007a; Piechowicz and Garbacz, 2016; Ben Said et al., 2017) also reported to lack genes for staphylococcal enterotoxins, suggesting a recent acquisition of the enterotoxin genes by CC15-MSSA isolates. Furthermore, while the MSSA isolates in this study belonged to ST15, the CC15-MRSA+SCC*fus* isolates in Kuwait (Udo et al., 2020) and Saudi Arabia (Raji et al., 2016; Senok et al., 2017) belonged to ST1535, a single locus variant of ST15, suggesting that the MRSA isolates emerged recently from ST15-MSSA isolates.

The high prevalence of CC361-MSSA in this study was different from the small numbers previously reported in patients in Kuwait hospitals (Vali et al., 2017) and in MSSA of animal origin in Egypt (El-Ashker et al., 2022) but mirrors the high prevalence of the CC361-MRSA isolates observed in patients in Kuwait hospitals (Boswihi et al., 2016; Sarkhoo et al., 2021). The majority of the CC361-MSSA isolates belonged to ST672, and the *spa* type t3841 was positive for *agrI, cap8,* and *egc* and was susceptible to most non-beta-lactam antibiotics similar to that of the majority of the CC361-MRSA isolates (Sarkhoo et al., 2021), suggesting a common origin for the CC361-MSSA and CC361-MRSA isolates in Kuwait hospitals.

The CC30-MSSA isolates belonged to ST30 and a novel ST8668, a single-locus variant of ST30, and 17 *spa* types. The dominant *spa* type, t021, was detected in both PVL-positive (*n* = 7) and PVL-negative (*n* = 3) isolates, similar to reports of CC30-MSSA, also known as phage 80/81 *S. aureus,* which was responsible for many outbreaks of infections in the 1950s and 1960s (DeLeo et al., 2011; McGavin et al., 2012; Deasy et al., 2019). The t012 isolates were similar to the majority of the CC30-MSSA isolates reported in Australia, the USA (DeLeo et al., 2011), and Nigeria (Essien et al., 2022).

The CC22-MSSA isolates belonged to a single ST, ST22. However, they belonged to 14 *spa* types, dominated by three *spa* types, t223, t005, and t309, indicating the diversity of the CC22-MSSA isolates. Furthermore, the isolates of t223 and t309 were positive for *tst1* and those belonging to t249, t309, t16061, and t005 were positive for PVL genes. The PVL-positive CC22-MSSA isolates have also been reported in other studies (Monecke et al., 2007b; Havaei et al., 2011; Yuan et al., 2019; Tayebi et al., 2020). The CC22-MSSA were resistant to benzylpenicillin and were susceptible to the majority of the non-beta-lactam antibiotics., were positive for *agrI, cap5, sak/chp/scn,* and the enterotoxin gene—egc—which mirrors the characteristics of the ST22-MRSA isolates obtained in Kuwait hospitals (Udo et al., 2016; Boswihi et al., 2022). Furthermore, the t249-CC22-MSSA isolates carried the genes for both PVL and *tst1* similar to the observations in recent CC22-MRSA isolates (Boswihi et al., 2022). The phenotypic and genotypic similarities between the CC22-MSSA and CC22-MRSA isolates are suggestive of their common origin.

The 22 CC398-MSSA isolates in this study are more than the two CC398-MRSA isolates obtained from human patients in Kuwait hospitals in 2016–2017 (Boswihi et al., 2020), suggesting that CC398-MSSA may be more common than CC398-MRSA in human patients in Kuwait. The CC398-MSSA comprising ST398, ST291, ST3629, and the novel STs, ST8660 and ST8666, all harbored *agr1* and *cap5,* and the majority was susceptible to non-beta-lactam antibiotics and lacked genes for enterotoxins similar to the characteristics reported previously in CC398-MSSA from humans (Bouiller et al., 2020) and animals (Feltrin et al., 2016). Furthermore, three ST291/ST813/t1149/t937 isolates were positive for genes encoding PVL, a characteristic associated with the human lineage (Bouiller et al., 2020). These observations suggest that the CC398-MSSA isolates in this study were of both animal and human origins.

The study also revealed that CC1, CC15, CC30, CC361, CC22, and CC88 were the dominant CCs in both study periods. However, while the number of CCs was higher in 2021 (*n* = 36) than in 2016 (*n* = 33), the distribution of the CC1, CC88, CC121, CC45, CC188, CC25, CC1223, and ST2867 isolates appeared relatively stable during the same period. On the other hand, the prevalence of CC15, CC361, CC30, and CC22-MSSA was lower in 2021 than in 2016. In contrast, some CCs were obtained sporadically, either in 2016 or 2021.

In conclusion, the study revealed a remarkable diversity in the clonal composition of the MSSA isolates. The MSSA isolates were more genetically diverse compared to the MRSA isolates, which were reported previously in Kuwait hospitals (Boswihi et al., 2016, 2020). Surprisingly, CCs that are dominant among MRSA in Kuwait were either detected sporadically or absent among the MSSA isolates except for CC1, CC15, and CC361, which were common among MSSA and MRSA isolates. The low prevalence of CC5 and ST239 lineages in this study when compared to their dominance among the previously reported MRSA population (Boswihi et al., 2020), suggests that they did not evolve from the local MSSA populations. The MSSA carried multiple virulence determinants similar to those previously reported in the MRSA isolates in Kuwait (Boswihi et al., 2016; Udo et al., 2017). The presence of multiple virulence factors in the MSSA isolates supports the notion that MSSA is as virulent as its MRSA counterparts (Rozgonyi et al., 2007). This study has also reported the first case of reduced susceptibility to daptomycin in *S. aureus* in Kuwait and highlighted the need for more investigations on the MSSA isolates to monitor changes in their resistance profiles and the distribution of emerging clones.

## Data availability statement

The original contributions presented in the study are included in the article/Supplementary material, further inquiries can be directed to the corresponding author.

## Author contributions

SB: Conceptualization, Formal analysis, Funding acquisition, Investigation, Methodology, Writing – original draft, Writing – review & editing. WA: Conceptualization, Formal analysis, Writing – original draft, Writing – review & editing. EU: Conceptualization, Formal analysis, Funding acquisition, Methodology, Supervision, Writing – original draft, Writing – review & editing.

## Footnotes

^1^https://pubmlst.org/organisms/staphylococcus-aureus
