# Corrigendum: Genomic profiling of methicillin-sensitive *Staphylococcus aureus* (MSSA) isolates in Kuwait hospitals

**DOI:** 10.3389/fmicb.2024.1507158

**Published:** 2024-10-18

**Authors:** Samar S. Boswihi, Wadha A. Alfouzan, Edet E. Udo

**Affiliations:** ^1^Department of Microbiology, College of Medicine, Kuwait University, Kuwait City, Kuwait; ^2^Microbiology Unit, Department of Laboratories, Farwaniya Hospital, Farwaniya, Kuwait

**Keywords:** MSSA, molecular genotyping, antibiotic resistance, *spa* types, DNA microarray

In the published article, there was an error in the Funding statement, where the funding numbers “PR17-MI12-02 from the Kuwait Foundation for the Advancement of Sciences (KFAS) and the Research Core Facility project no. SRUL02 from Kuwait University, Research Sector” were wrong.

The correct Funding statement appears below.

